# SVF Combined with HGF-Functionalized Self-Assembling Peptide Hydrogel Promotes Spinal Cord Injury Repair in Rats

**DOI:** 10.3390/gels12070638

**Published:** 2026-07-16

**Authors:** Feng Yang, Tiantian Li, Yu Wang, Yanling Chen, Xuhuai Chen, Linshu Ding, Yuanyi Liu, Jialin Li, Guanbo Huang, Haibo Zhou, Qiuju Yuan, Wutian Wu

**Affiliations:** 1 Guangdong-Hong Kong-Macau Institute of CNS Regeneration, College of Life Science and Technology, Jinan University, Guangzhou 510632, China; 2 Re-Stem Biotechnology Co., Ltd., Suzhou 215129, China

**Keywords:** spinal cord injury, stromal vascular fraction, cell transplantation, hydrogel scaffold, combinatorial therapy

## Abstract

Spinal cord injury (SCI) is a devastating neurological disorder. The development of effective therapies to ameliorate the consequences of SCI represents a major challenge and a central priority of international biomedical research. The stromal vascular fraction (SVF) derived from adipose tissue possesses considerable functional potential. SVF is a heterogeneous mixture of cells that act synergistically. However, after local transplantation, SVF is rapidly cleared via the bloodstream, and its poor survival severely compromises therapeutic efficacy. To overcome this limitation, we employed a self-assembling peptide nanohydrogel HGF-RADA16-IKVAV (where HGF denotes the tripeptide histidine–glycine–phenylalanine) as a scaffold to enhance SVF retention and efficacy in a rat model of SCI. Implantation of SVF and HGF into the injured spinal cord demonstrated that this combined therapy significantly modulated the inflammatory response, increased neuronal survival, and promoted a denser network of axon tracts. Consequently, the SVF-encapsulated HGF hydrogel resulted in superior restoration of limb movement and reduced neuropathic pain. Proteomic analysis confirmed that the combined treatment shifted the injury-induced molecular landscape, particularly in immune and inflammatory pathways. Collectively, these findings demonstrate that this combinatorial strategy represents an effective therapeutic paradigm for SCI.

## 1. Introduction

Spinal cord injury (SCI) can occur after several external agents, direct or indirect, that cause compression, rupture or deformation of the spinal cord. This is a serious neurological disorder that can lead to severe impairment of physical function. The injury causes loss of movement and sensation below the level of injury [1]. SCI is a disabling condition with significant implications for the physical and mental health of patients [2], leading commonly to paralysis of different degrees. SCI pathophysiology is a complex and dynamic process, classically divided into primary and secondary injury mechanisms [3]. SCI results in architectural disruption of the spinal cord and a phase-dependent inflammatory response. In the acute phase, there is infiltration of neutrophils and activation of resident microglia, leading to an exacerbated tissue injury by the production of pro-inflammatory cytokines (e.g., TNF-α, IL-1β), ROS, and proteolytic enzymes [4]. In the sub-acute and chronic phases, the formation of glial scars is caused by the chronic activation of microglia and macrophages and the proliferation of astrocytes [5]. The hostile microenvironment is a significant obstacle to endogenous repair and therapeutic interventions. Recent advances in biomaterials and tissue engineering have led to the emergence of new therapeutic strategies for SCI in recent decades [6,7]. A self-assembling peptide hydrogel, HGF-RADA16-IKVAV (HGF) (where HGF denotes the tripeptide histidine–glycine–phenylalanine), was used here. The N-terminus is functionalized with histidine (imidazolium group), glycine, and phenylalanine (aromatic group). HGF is a tripeptide (HGF: histidine–glycine–phenylalanine). RADA16-I is a peptide with four repeats of R (arginine), A (alanine), D (aspartic acid), and A (alanine), and the C-terminus of the RADA16-I self-assembling oligopeptide can bind to IKVAV (isoleucine–lysine–valine–alanine–valine) [5]. This change improves the scaffold’s adhesion to the wet spinal cord tissue, thus favoring its retention at the injury site [8]. Along with the development of biomaterials, cell transplantation therapies have been widely investigated for SCI [9,10]. Adipose-derived SVF is a viable and effective cellular therapy. Its application involves temporomandibular joint repair, promotion of gastrointestinal regeneration, and amelioration of acute myocardial infarction [11,12,13]. SVF is a mixture of different cell types isolated from adipose tissue lipoaspirate by enzymatic digestion [14]. SVF cells have been shown to produce a variety of trophic factors (e.g., BDNF, GDNF, and VEGF) that promote neuroprotection, angiogenesis, and axonal sprouting [15,16,17]. In addition, SVF has a strong immunomodulatory effect on the suppression of adverse neuroinflammation [18]. SVF is sourced from the patient’s own tissue, thereby circumventing the immune rejection problems seen with allogeneic grafts and the ethical issues surrounding pluripotent stem cell-based therapies. We postulated that HGF hydrogel could be an ideal bioactive scaffold to directly entrap and retain the SVF cells in the SCI lesion. Here, we tested the hypothesis that augmenting survival of SVF in the injured spinal cord with the HGF hydrogel would provide a persistent local source of trophic support and immunomodulation leading to more effective modification of the injury microenvironment, greater tissue preservation, and ultimately better functional recovery than either component alone. To dissect the underlying molecular mechanisms, we performed a systematic evaluation of this combinatorial therapy by longitudinal behavioral analyses, comprehensive histological assessment, and unbiased proteomic analysis. The findings of our study indicate that combined SVF + HGF therapy enhances motor function in the hind limb, suppresses inflammation at the SCI lesion site, remodels the local glial and inflammatory microenvironment, and promotes neuronal survival and nerve fiber regeneration. This is the first systematic evaluation of the SVF + HGF hydrogel in a preclinical SCI model and has novel findings that have not been previously reported. This hydrogel can not only provide a scaffold for cells but also build a suitable microenvironment for the survival and proliferation of SVF cells. The results provide a basis for further systematic studies and therapeutic strategies by transplanting neural stem cells with hydrogels for treating injuries of the central nervous system.

## 2. Results and Discussion

### 2.1. The HGF Hydrogel Serves as a Potent Scaffold for SVF Retention

**Figure 1:**
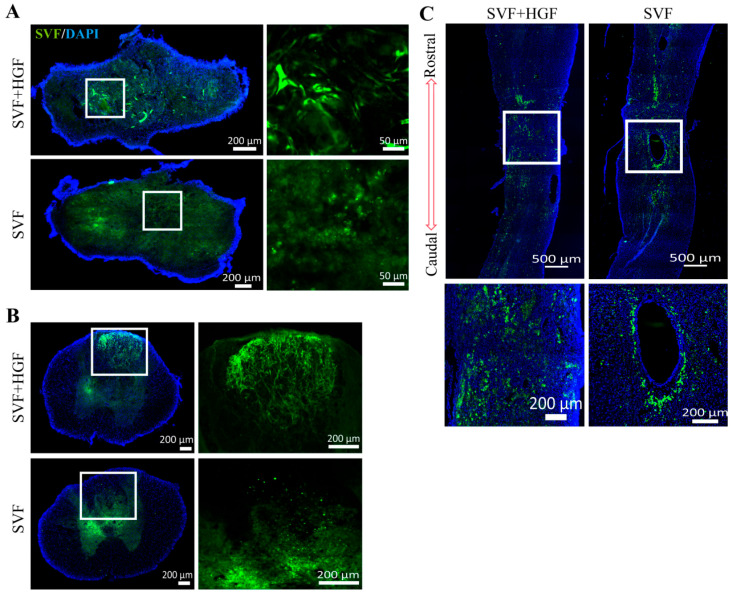
Migration of SVF cells after transplantation. (**A**,**B**) Migration of SVF cells at 14 days post-transplantation in the SVF + HGF group and the SVF group: (**A**) SVF migration in the central region of SCI; (**B**) SVF migration in the spinal cord at 3 mm rostral to the SCI site. (**C**) Migration of SVF cells at 56 days post-transplantation in the SVF + HGF group and the SVF group. *n* = 3 for each group. SVF (green), DAPI (blue).

One of the main issues of this study was whether SVF could be retained in the SCI lesion following loading in HGF hydrogel. GFP+ cells survived in the center of the SCI site in the SVF + HGF group after 14 days of transplantation with intact cell morphology under the influence of the hydrogel. At 3 mm rostral to the center of the lesion, SVF cells were mainly localized in the dorsal regions of the spinal cord with an aggregated cellular morphology (Figure 1A,B). This suggested that SVF cells have the capacity to migrate to the vicinity of the center of SCI. On the other hand, in the SVF group, the GFP signal was weak and widely distributed throughout the spinal cord parenchyma, often several millimeters away from the site of injection, indicating rapid and extensive cell loss in the absence of a scaffold. At 56 days, the number of GFP+ cells dropped in both groups, as expected with cell turnover and phagocytosis. At the 56-day endpoint of the experiment, SVF cells were still present in two groups (Figure 1C).

### 2.2. Combinatorial Therapy Driven by Enhanced Retention Promotes Robust Functional Recovery

**Figure 2:**
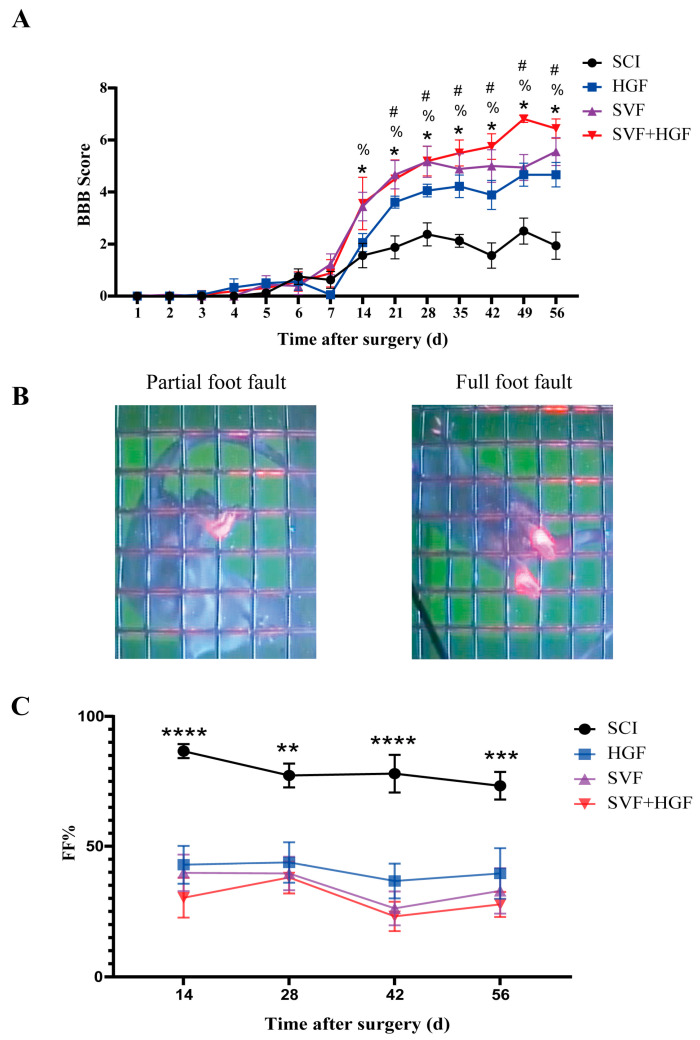
SVF and HGF improve motor function in rats. (**A**) BBB scores over 8 consecutive weeks post-SCI. (**B**,**C**) Foot slip test in rats after SVF + HGF transplantation. (**B**) Schematic of partial vs. complete foot faults. (**C**) Statistical analysis of foot fault rate (FF%) in SCI, HGF, SVF, and SVF + HGF groups. At each time point, statistical comparisons among the four groups were performed using one-way ANOVA with Tukey’s post hoc test. Data are expressed as mean ± SD (*n* = 6). * *p* < 0.05 versus the SCI group. ** *p* < 0.01 versus the SCI group. *** *p* < 0.001 versus the SCI group. **** *p* < 0.0001 versus the SCI group. % *p* < 0.05 versus the HGF group. # *p* < 0.05 versus the SVF group.

We next investigated the functional consequences of this increased cell retention. The SVF + HGF group showed a significantly faster and more complete recovery of locomotor function. The scores of this group started to diverge from the other groups on the BBB scale at day 14, and from day 21 through the end of the experiment at day 56, the scores were statistically significantly better than the SCI, HGF, and SVF groups (*p* < 0.05) (Figure 2A). The SVF and HGF groups also improved over the SCI group, but the recovery plateaued at a lower level, emphasizing the synergistic effect of the combination. These findings were confirmed by the foot slip test, which is a test of finer coordination and proprioception. The SVF + HGF group committed significantly fewer errors in total and had a lower foot fault rate than all other groups (*p* < 0.001) from day 14 onwards (Figure 2B,C). This indicates that the combinatorial therapy restored not only the gross locomotor movement but also significantly improved the precision of limb placement.

**Figure 3:**
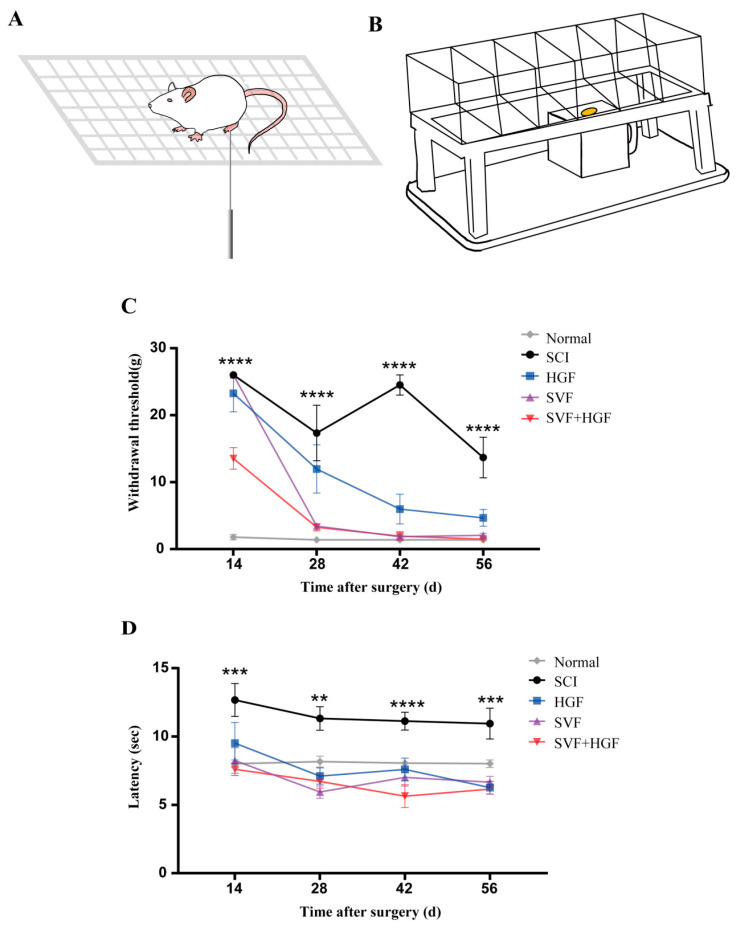
Analysis of mechanical and thermal pain in rats after SVF + HGF transplantation. (**A**) Schematic of mechanical allodynia testing: rats undergo mechanical pain threshold assessment via Von Frey filaments on a grid. (**B**) Schematic of thermal hyperalgesia testing: rats are placed on a hot plate to observe paw-withdrawal or paw-licking response times. The yellow dots represent a radiant heat source lamps used for thermal pain testing. (**C**) Mechanical pain sensitivity testing in normal, SCI, HGF, SVF, and SVF + HGF groups. (**D**) Thermal pain sensitivity testing in normal, SCI, HGF, SVF, and SVF + HGF groups. Statistical comparisons among groups were performed using one-way ANOVA with Tukey’s post hoc test. Data are expressed as mean ± SD (*n* = 6). ** *p* < 0.01 versus the SCI group. *** *p* < 0.001 versus the SCI group. **** *p* < 0.0001 versus the SCI group.

Neuropathic pain, a common and debilitating sequela of SCI, was also studied. Mechanical allodynia and thermal hyperalgesia were significantly attenuated in the treatment groups by postoperative day 28. The SVF and SVF + HGF groups showed the most significant increase in the mechanical pain thresholds (*p* < 0.001 vs. SCI), and the SVF + HGF group also showed a significant increase in the thermal withdrawal latency (*p* < 0.001) (Figure 3).

### 2.3. Functional Recovery Occurs Without Corticospinal Tract (CST) Regeneration

**Figure 4:**
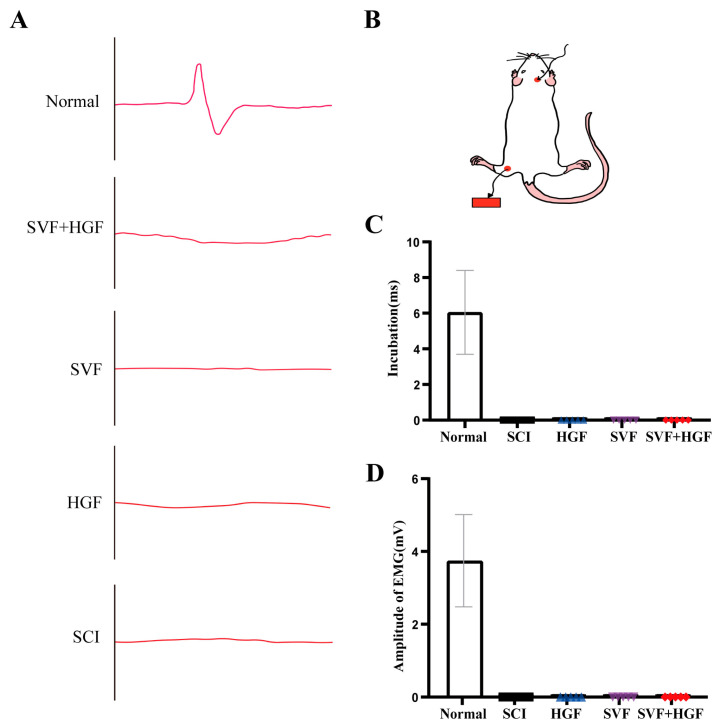
Electromyography in rats after SVF + HGF transplantation. (**A**) Action potential recordings in the normal, SCI, HGF, SVF, and SVF + HGF groups. (**B**) Schematic of the electromyography experiment: electrical stimulation is applied to the motor cortical region of rat brains, with signal reception at the contralateral hind limb gastrocnemius muscle. (**C**) Comparison of electromyographic amplitude across the 5 groups. (**D**) Comparison of EMG latency among the 5 groups. Statistical comparisons were performed using one-way ANOVA with Tukey’s post hoc test. Data are expressed as mean ± SD (*n* = 5).

Despite the marked behavioral recovery, cortical stimulation did not evoke gastrocnemius muscle motor evoked potentials (MEPs) in any SCI cohort at the 56-day time point (Figure 4). This indicated that the functional benefits were not mediated by long-distance CST regeneration. Instead, the recovery may have been attributable to neuroprotection of spared circuits and intraspinal reorganization of proprio-spinal interneurons to relay signals from intact corticospinal axons above the lesion.

### 2.4. A Modulated Inflammatory Microenvironment and Enhanced Tissue Preservation

**Figure 5:**
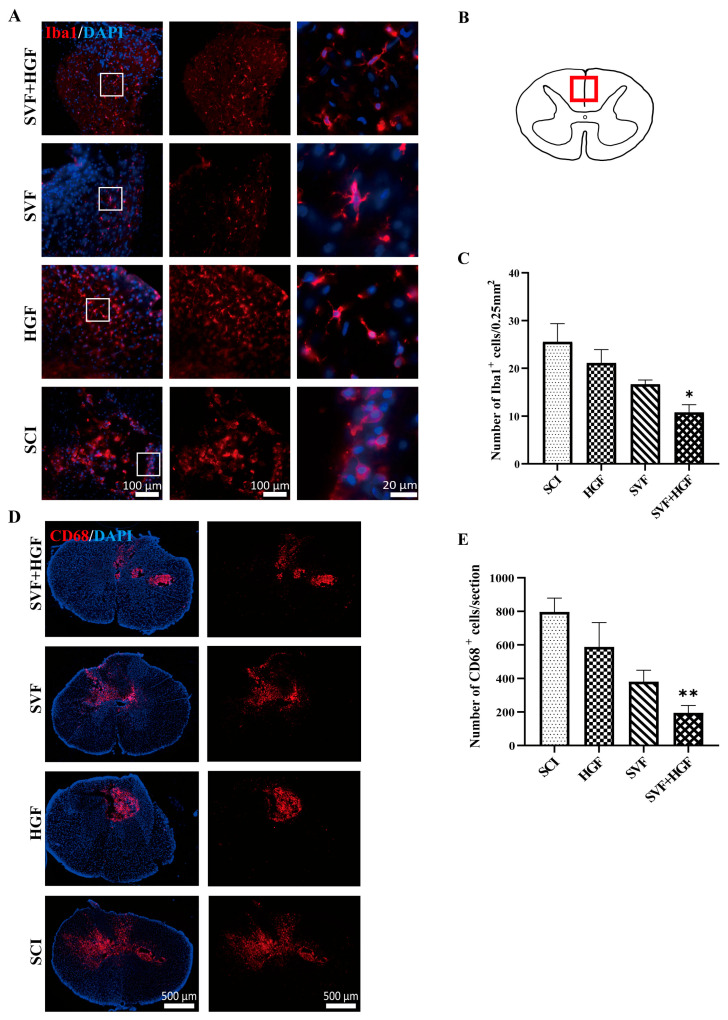
SVF + HGF transplantation suppresses the inflammatory response after SCI. (**A**) Number and morphology of Iba1^+^ microglia in the dorsal spinal cord at 4 mm rostral to the SCI site among the experimental groups. (**B**) Schematic diagram of the quantification region for Iba1^+^ microglia count at 4 mm rostral to the SCI site. (**C**) Statistical analysis of Iba1^+^ cells across four groups. (**D**) The number and morphology of CD68^+^ microglia in the dorsal spinal cord, located 3 mm rostral to the SCI site, across the four experimental groups. (**E**) Statistical analysis of CD68^+^ cells across four groups. Statistical comparisons were performed using one-way ANOVA with Tukey’s post hoc test. Data are expressed as mean ± SD (*n* = 3). * *p* < 0.05 versus the SCI group. ** *p* < 0.01 versus the SCI group. Iba1 (red), CD68 (red), DAPI (blue).

To identify the histological correlates of the functional recovery, we examined key cellular players in the injury response. At 14 days post-injury, the peak of the secondary damage cascade, the SVF + HGF group showed a significant reduction in neuroinflammation. Compared with the SCI group, the number of activated Iba1^+^ microglia in the dorsal anterior region of the injured spinal cord was reduced, and these cells exhibited larger, amoeboid morphology (*p* < 0.05) (Figure 5A–C). Furthermore, the number of CD68^+^ phagocytic macrophages was markedly lower in the SVF + HGF group than in both the SCI and SVF groups (*p* < 0.01) (Figure 5D,E).

**Figure 6:**
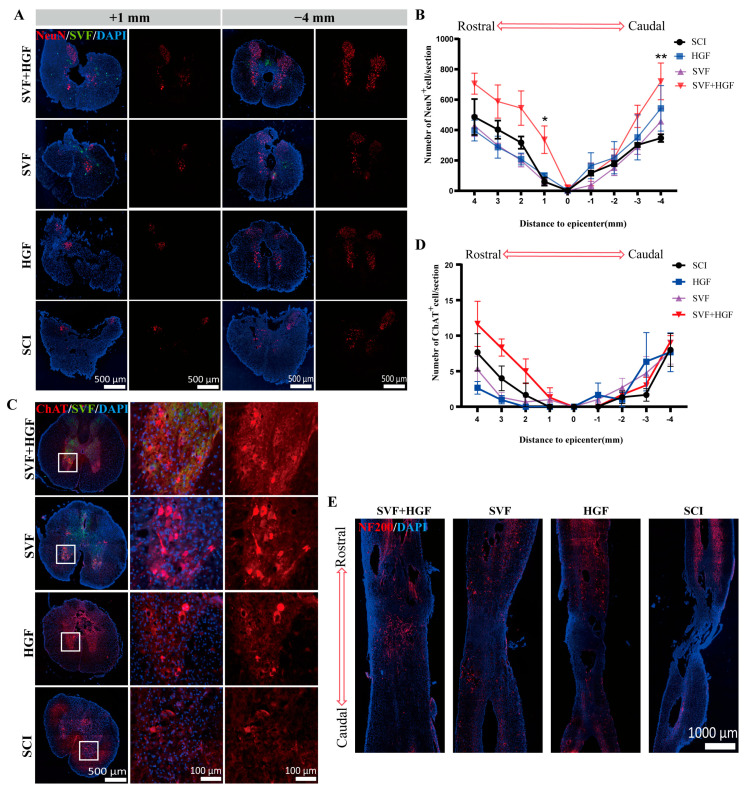
SVF + HGF transplantation in rats promotes neuronal survival and nerve fiber regeneration at 56 days post-transplantation. (**A**) NeuN^+^ neuron staining among the four groups. (**B**) Statistical analysis of NeuN^+^ neurons among the four groups. (**C**) ChAT^+^ neuron staining among the four groups. (**D**) Statistical analysis of ChAT^+^ neurons among the four groups. (**E**) NF200^+^ nerve fiber staining in the four experimental groups. Statistical comparisons were performed using one-way ANOVA with Tukey’s post hoc test. Data are expressed as mean ± SD (*n* = 3). * *p* < 0.05 versus the SCI group. ** *p* < 0.01 versus the SCI group. NeuN (red), ChAT (red, NF200 (red), SVF (green), DAPI (blue).

This less hostile milieu was conducive to tissue preservation. At 56 days, the SVF + HGF group had significantly more surviving NeuN^+^ neurons at multiple segments (1 mm rostral and −4 mm caudal to the epicenter) than any other group (*p* < 0.05) (Figure 6A,B). Moreover, an increase in the survival of ChAT^+^ motor neurons was observed in the anterior horn (Figure 6C,D). NF200 staining showed a much denser network of neurofilament-positive fibers within and passing through the lesion site in the SVF + HGF group, which is consistent with increased axonal sparing or active regeneration/sprouting (Figure 6E).

### 2.5. Proteomic Analysis Confirms a Global Shift Towards a Repair-Conducive State

**Figure 7:**
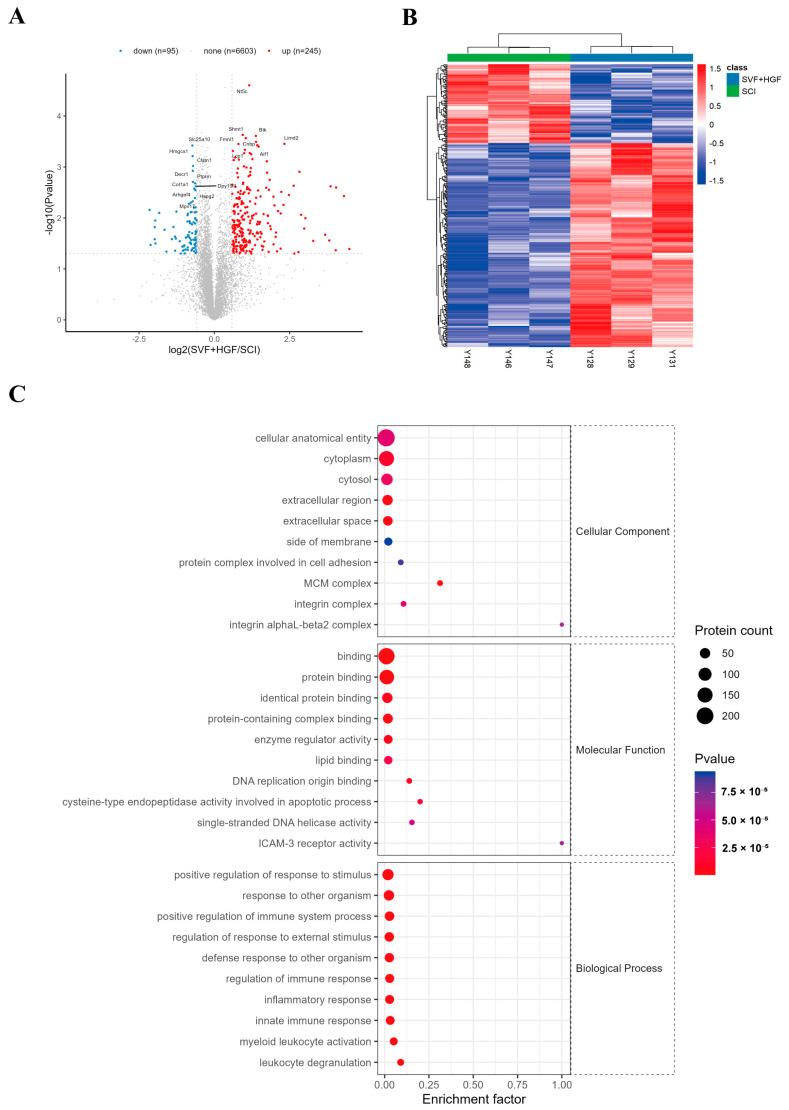
Altered protein profiles in rat spinal cords following SVF + HGF implantation at 2-week intervals. (**A**) Volcano plot of proteins identified via LC-MS analysis. Red dots signify increased protein expression; blue dots denote decreased protein expression. (**B**) Heatmap showing the distribution of differentially expressed proteins. High expression is indicated by red, while low expression is represented by blue. (**C**) Findings derived from GO enrichment analysis with respect to biological processes.

**Figure 8:**
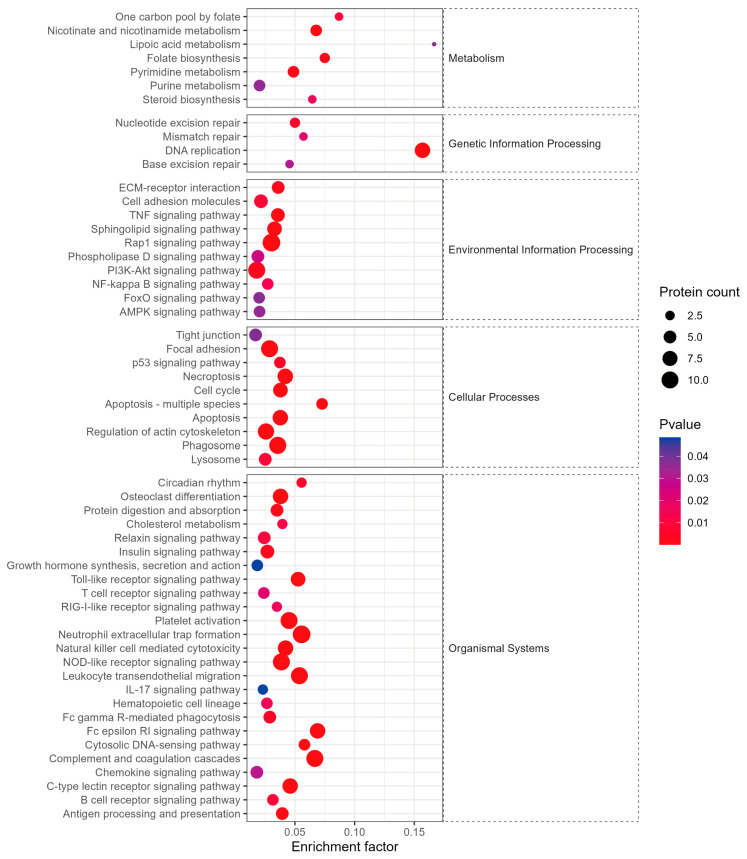
KEGG pathway enrichment analysis of differentially expressed proteins. Shown are KEGG pathways significantly enriched among the differentially expressed proteins between the SVF + HGF and SCI groups. Enrichment factor, protein count, and *p*-values are indicated for each pathway. Pathways related to metabolism, nucleotide repair, and cell signaling were among those identified.

The quality of the proteomics data was first assessed by examining peptide and protein identification counts, which were comparable across all groups, confirming data reliability (Supplementary Figure S2). We then performed DIA proteomic analysis of peri-lesion tissue at 14 days to provide an unbiased systems-level view of the treatment effect. We found 340 differentially expressed proteins between the SVF + HGF and SCI groups, 245 upregulated and 95 downregulated. Principal component analysis (PCA) of global protein expression profiles revealed clear separation among the Control, SCI, and SVF + HGF groups (Supplementary Figure S3). The two principal components (PC1 and PC2) explained 66.05 and 9.78% of the total variance, respectively. Control samples congregated at the negative end of PC1 with consistent baseline protein expression. SCI samples had a wider distribution, indicating the heterogeneous injury response. Notably, SVF + HGF samples moved toward the control group on PC1, indicating that the combined treatment partially restored the protein expression profile altered by the injury. Proteomic screening identified five key DEPs associated with spinal cord microenvironment remodeling following SVF + HGF hydrogel transplantation. Compared with the SCI group, PIK3R1 and AKT1 were moderately upregulated in the SVF + HGF group, while the pro-inflammatory cytokines IL-1β, TNF-α, and microglia/macrophage activation marker CD68 were markedly downregulated (Figure 7A). These DEPs were mainly enriched in neuroinflammation and PI3K-Akt cell survival pathways (Figure 8). Volcano plot visualization (Figure 7A) highlighted the molecular changes induced by the therapy. GO analysis of these proteins showed a highly significant enrichment of terms related to “immune system process,” “inflammatory response,” “innate immune response,” and “defense response” (Figure 7C). This molecular signature is consistent with our histological findings of decreased neuroinflammation. KEGG pathway enrichment analysis further revealed the participation in critical signaling pathways like “Phagosome” and “PI3K-Akt signaling pathway,” a well-known stimulator of cell survival and growth [19] (Figure 8).

**Figure 9:**
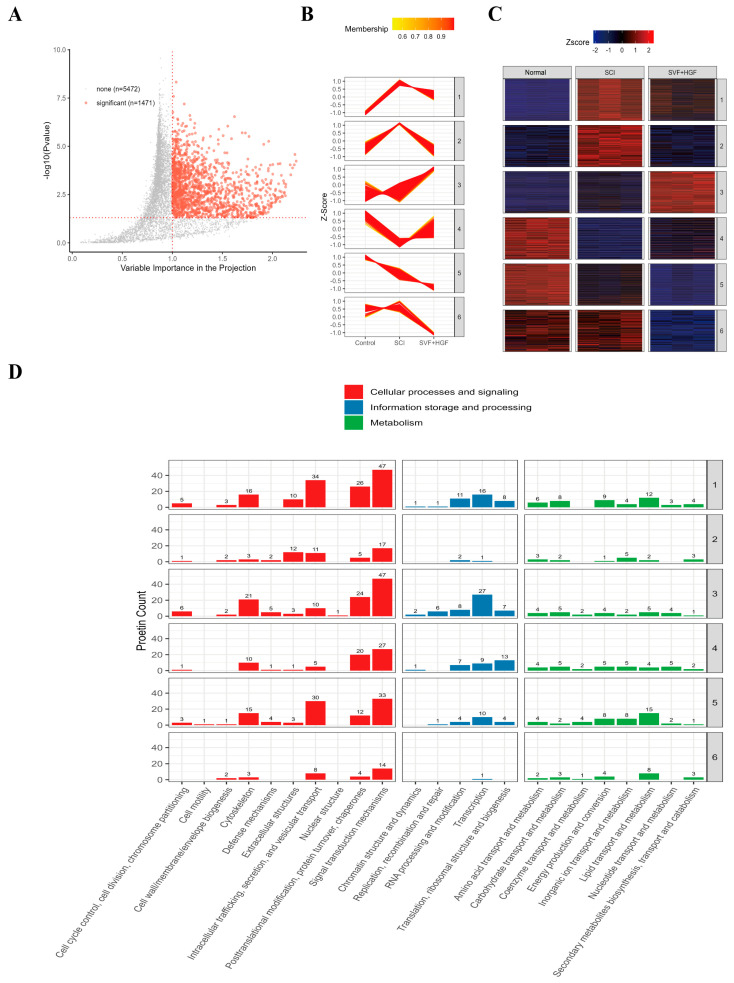
Multi-phenotype differential analysis. (**A**) Multi-phenotype differential indicator analysis. (**B**–**D**) K-means clustering analysis of multi-phenotype differential proteins. (**B**) Expression trend plot. (**C**) Protein heatmap. (**D**) Functional entry statistical analysis plot. *n* = 3 for each group.

Most compellingly, multi-phenotype clustering analysis revealed that the global protein expression profile of the SVF + HGF group was shifted away from the diseased state of the SCI group and closer to that of an uninjured normal group (Figure 9). This suggests that the combinatorial treatment induced a global “normalization” of the molecular microenvironment, reverting many of the negative changes initiated by the injury and creating a state more favorable to repair.

### 2.6. Discussion

The present study results support our major hypothesis that combined transplantation of SVF + HGF accelerates functional recovery after SCI. This combinatorial strategy successfully suppressed the pathological inflammatory response, protected neurons and axons, improved hind limb motor function, and alleviated neuropathic pain compared to SVF alone, hydrogel alone, or untreated controls. The hydrogel scaffold plays a pivotal role in overcoming the critical limitation of poor SVF retention after local transplantation. Our data show that the HGF matrix prevents rapid dispersion of SVF cells, enabling them to fully exert their paracrine potential. SVF cells are known to secrete IL-10 and TGF-β [20,21], and the hydrogel may create a niche that supports such secretion. These factors are critical for establishing a microenvironment that protects host neural cells from secondary degeneration after SCI. Consistent with the known properties of injectable hydrogels as ECM-mimetic scaffolds [22,23,24,25], the HGF matrix provides three-dimensional support, fills irregular lesion cavities, and facilitates cell migration and axonal growth [26,27,28,29,30,31].

Nevertheless, successful engraftment remains a major hurdle for all CNS cell therapies, including SVF-based approaches [20,32]. The acute SCI microenvironment is highly hostile to cell survival and engraftment due to inflammation, excitotoxicity, and ischaemia [33,34,35,36]. Our data show that the SVF + HGF combination directly counteracts this hostile environment. The local paracrine effect of the retained SVF cells sufficiently inhibited acute inflammatory responses, as evidenced by the significant reduction in activated Iba1+ microglia and CD68^+^ macrophages at the lesion site and its periphery. This histological finding is well supported by global proteomic analysis.

Proteomic data of differential expression of proteins revealed a coordinated molecular response to SVF + HGF treatment. Upregulation of PIK3R1 and AKT1 indicated activation of the PI3K-Akt pro-survival signaling pathway, and downregulation of IL-1β, TNF-α and CD68 indicated reduced neuroinflammation and microglial/macrophage activation [37,38]. The volcano plot further illustrated the magnitude of these molecular changes. Consistent with these results, GO enrichment analysis identified significant over-representation of terms related to “immune system process” and “inflammatory response”, directly supporting our histological findings of reduced neuroinflammation. Further KEGG pathway analysis indicated “Phagosome” and “PI3K-Akt signaling pathway” as major enriched pathways, with the latter being a known promoter of cell survival and growth [19]. Together, these data suggest that the combined treatment promotes a shift from a pro-inflammatory, injury-associated state toward a more repair-permissive microenvironment, thereby supporting both neuroprotection and functional recovery.

Most convincingly, multi-phenotype clustering analysis provided the most convincing evidence: the global protein expression profile of the SVF + HGF group shifted away from the injured state of the SCI group and towards that of uninjured normal tissue. This indicates a widespread “normalization” of the molecular microenvironment towards a repair-permissive state.

Based on these molecular and histological findings, early inhibition of secondary injury mechanisms is critical to prevent widespread apoptosis of neurons and oligodendrocytes. Accordingly, we observed significantly increased survival of NeuN^+^ and ChAT^+^ motor neurons and a denser preserved network of NF200^+^ axons in the SVF + HGF group. This is consistent with a mechanistic cascade in which improved cell retention leads to enhanced paracrine signaling, driving effective immunomodulation, superior neuroprotection and tissue sparing, and ultimately the observed functional recovery. Thus, the SVF + HGF hydrogel system represents a promising strategy to engineer a pro-regenerative local microenvironment after SCI.

Turning to functional outcomes, recovery of hind limb motor function, as measured by BBB scoring and the foot slip test, is likely attributable to plasticity of surviving neural circuits rather than regeneration of long-distance tracts. This interpretation is supported by the absence of motor evoked potentials (MEPs) across the lesion, indicating no functional regeneration of the corticospinal tract. Instead, improved locomotor function is likely to be mediated by plasticity of propriospinal interneurons, sparing of descending monoaminergic pathways, and reorganization of local spinal circuits [39,40,41]. Interestingly, the significantly higher number of surviving interneurons (NF200^+^, NeuN^+^, and ChAT^+^ cells) in the SVF + HGF group at day 56 (Figure 6) suggests that the treatment may help to preserve interneuron populations that can act as relay elements, allowing residual signal transmission across the lesion site [42,43,44]. The therapy may also encourage reorganization of intraspinal circuitry, such as propriospinal interneurons, to relay information across or around the injury site [43,45,46]. Furthermore, the reduction in mechanical and thermal hypersensitivity suggests that the treatment also modulates the maladaptive hyperexcitability of spinal sensory circuits involved in neuropathic pain. Together, these data support the conclusion that neuroprotection and plasticity of spared systems can lead to substantial functional recovery without requiring long-tract regeneration.

Beyond its role as a passive scaffold, the HGF hydrogel itself actively contributes to this process. The IKVAV epitope is known to promote neurite adhesion and outgrowth, and hydrogels of similar composition have been shown to enhance synaptic formation and remyelination in SCI models [47]. The hydrogel likely provides a permissive ECM-mimetic matrix that supports local axonal sprouting and reorganization of neural connections. In addition to these mechanistic insights, our combinatorial approach offers unique translational advantages. SVF is an autologous cell product derived from adipose tissue—clinically accessible, free from major ethical concerns, and associated with fewer immunological complications [48,49,50]. The HGF hydrogel is a synthetic, chemically defined, biocompatible material. Their synergy overcomes the major limitation of each component: the hydrogel provides the spatial retention and topographical cues that SVF cells require, while SVF cells supply a broad array of bioactive signals that the hydrogel alone cannot replicate. Together, they form a complete therapeutic system that is more effective than either component alone.

Potent therapeutic efficacy of autologous SVF transplantation has been demonstrated in preclinical CNS injury models, but several translational challenges, including technical limitations in isolation, significant inter-batch variability and limited scalability, impede its routine clinical application. The manufacturing process is constrained by procedural risks associated with fat harvest in acutely injured patients, dependence on xenogeneic reagents that are not GMP-compliant and use of closed animal-free systems that reduce cell yield. SVF composition and paracrine function vary greatly with donor age, inflammation, and metabolic status. Lack of standardized potency release criteria results in inconsistent treatment responses. The SVF is patient-specific and not scalable; therefore, the SVF must be used at the bedside immediately within a narrow therapeutic window. Native SVF cannot be expanded without loss of key functional properties. While some solutions are emerging to tackle these challenges, such as closed automated processing systems, standardized potency assays and short-term progenitor amplification, rigorous GMP workflow optimization and uniform product characterization are critical to translate preclinical promise into consistent clinical outcomes.

### 2.7. Limitations

Some limitations of this study need to be acknowledged. First, the contusion/compression model is clinically relevant but does not fully capture the complexity and heterogeneity of human SCI. In addition, the present study was restricted to acute intervention, and the delineation of the therapeutic window and assessment of efficacy at subacute or chronic phases of injury are important next steps for clinical translation.

Several additional limitations should be acknowledged. First, we did not perform quantitative analysis of lesion volume, cystic cavity size, or tissue sparing—parameters commonly reported in spinal cord injury studies that would provide important structural correlates of recovery. Second, the histological and proteomic analyses were performed with a relatively small sample size (*n* = 3 per group). Although this is consistent with exploratory studies in the field and our statistical analyses revealed significant differences for several key endpoints, the limited sample size may reduce statistical power and generalizability. Independent replication in larger cohorts is warranted to confirm these findings.

Hydrogel characterization was not performed by measuring quantitative rheological parameters independently in this study since this material was well characterized in our previous work [8,51]. Similarly, long-term in vivo degradation kinetics, hydrogel persistence and cell survival beyond 56 days were not evaluated. Further studies will include long-term in vivo tracing and full biosafety analysis.

Similarly, while several key differentially expressed proteins were identified by the proteomic analysis, these findings are exploratory and were not independently validated by orthogonal methods such as Western blotting, ELISA, or targeted parallel reaction monitoring (PRM). Although our PCA and quality control analyses (Supplementary Figures S2 and S3, mass accuracy < 10 ppm, peptide FDR < 1%, protein FDR < 1%) support the reliability of the dataset, independent validation of key protein candidates will be necessary to confirm the biological relevance of these changes. The proteomics analysis was performed on a limited number of samples (*n* = 3 per group), and therefore the results are hypothesis-generating rather than confirmatory. Future studies with larger cohorts and orthogonal validation will be required to substantiate these findings.

## 3. Conclusions

In conclusion, we have established a transplantation strategy for SCI using autologous adipose-derived SVF in combination with HGF. This strategy effectively attenuates post-traumatic neuroinflammation, enhances neuronal and axonal preservation, and promotes hind limb motor recovery with concurrent relief from neuropathic pain. The hydrogel scaffold solves the major limitation of poor retention of SVF, which allows sustained local paracrine effects and immunomodulation. SVF has unique advantages over single-stem-cell therapies in modulating the injury microenvironment and supporting neuronal survival. This simple yet powerful concept addresses a fundamental flaw of cell therapy and leads to robust microenvironmental modulation, substantial tissue preservation and meaningful functional recovery. Our results provide a strong preclinical basis for the clinical translation of this synergistic cell-scaffold therapy for SCI.

## 4. Materials and Methods

### 4.1. Animals and Ethical Statement

All animal experiments were approved by the Laboratory Animal Ethics Committee at Jinan University, China (approval no. IACUC-20251031-06). Female Sprague-Dawley rats (8–10 weeks old, 220–250 g) were purchased from the Guangdong Medical Experimental Animal Center (license no. SYXK (Yue) 2022-0174). A total of 84 animals were randomly allocated to four groups of 20 animals each. Exclusion criteria included dural rupture and intraoperative death. Final sample sizes were as follows: behavioral studies, *n* = 6 per group; histology (day 14 and day 56), *n* = 3 per group per time point; electrophysiology, *n* = 5 per group; and proteomics, *n* = 3 per group. For SVF isolation, GFP-transgenic Sprague-Dawley rats (on the same genetic background) served as donors to enable cell tracking. All methods were carried out in accordance with relevant guidelines and regulations and reported in accordance with ARRIVE guidelines [52]. The detailed animal allocation, exclusions, and final sample sizes are summarized in the CONSORT-like experimental flow diagram (Supplementary Figure S1) and the sample size table (Supplementary Table S2).

### 4.2. Preparation of Adipose-Derived SVF

Anesthesia was induced by intraperitoneal injection of 1% sodium pentobarbital (32 mg/kg). SVF was harvested from the inguinal adipose tissue of each GFP-transgenic rat under anesthesia. SVF was isolated from Sprague Dawley rats transgenic for GFP (ubiquitously expressing EGFP under the CAG promoter) [51], which was used as donors for long-term cell tracking in vivo. Excess tissue was trimmed away, and the adipose tissue was cut into small pieces using ophthalmic scissors. It was extensively washed with several washes of Hank’s Balanced Salt Solution (HBSS, Gibco, Grand Island, NY, USA) sterile solution with 1% penicillin-streptomycin. The tissue fragments were enzymatically digested at 37 °C for 60 min with gentle agitation in 0.1% type I collagenase (Sigma-Aldrich, St. Louis, MO, USA). To inhibit enzymatic activity, an equal volume of Dulbecco’s modified Eagle medium (DMEM) containing 10% fetal bovine serum (FBS) was added. Undigested tissue fragments were removed by sequential filtration of the cell suspension through 100 μm strainers. The filtrate was centrifuged, and the supernatant was collected. The pelleted SVF cells were reconstituted in HBSS to a final concentration of 2 × 10^4^ cells/μL for immediate use in transplantation. The cellular composition of the SVF from rats has been studied several times before by flow cytometric analysis using the identical isolation protocol [51] in our lab, the results are quite consistent.

### 4.3. Preparation and Characterization of HGF Hydrogel

The HGF (2%) hydrogel was prepared by dissolving the peptide in sterile deionized water. The peptide (H_2_N-RADA16-IKVAV-GG-HGF-CONH_2_; HGF = His-Gly-Phe) had an amide group on the N-terminal [51]. The solution was passed through a 0.22 μm syringe filter under sterile conditions, and the pH was adjusted to 7.4. The stable nanofibrous hydrogel was quickly self-assembled within 30 min at room temperature. The HGF-RADA16-IKVAV self-assembling peptide hydrogel was prepared as previously described [8,51].

The self-assembling peptide hydrogel (HGF-RADA16-IKVAV) was purchased from custom synthesis, China Peptides Co., Ltd. (Shanghai, China) and prepared according to the manufacturer’s instructions. Key physicochemical properties are summarized in Supplementary Table S1. The mechanical properties and nanofibrous structure of the hydrogel were confirmed by rheometry and scanning electron microscopy in our previous work [8]. Briefly, the hydrogel exhibits a storage modulus (G′) of 800 Pa and a loss modulus (G″) of 150 Pa. Regarding degradation behavior, while the in vivo degradation kinetics of this specific hydrogel formulation were not quantitatively characterized in the present study, the hydrogel was prepared fresh for each experiment, and batch-to-batch variability was minimal as verified by consistent gelation time and mechanical properties across batches. Future studies will be required to systematically evaluate the in vivo degradation profile of this hydrogel in the spinal cord injury microenvironment.

### 4.4. SCI Model

A complete SCI model was established at the level of the T9 vertebral body as described previously [53]. Rats were induced into a deep anesthetic state via intraperitoneal injection of 1% pentobarbital sodium (32 mg/kg). The paravertebral muscles were carefully dissected after a dorsal midline incision was performed so as to expose the T9–T10 vertebral levels. A T9 laminectomy was performed to completely reveal the spinal cord beneath. A complete spinal cord clamp injury was then induced using No. 5 Dumont forceps (Fine Science Tools, Foster City, CA, USA tip width 0.5 mm) applied extradurally for 3 s. This clamping procedure was repeated 3 times with 3 min intervals to ensure a severe and consistent injury. The repeated clamp injury (3 compressions with 3 min intervals) was chosen to produce a severe and reproducible complete crush injury with minimal variability, as described previously [53,54,55]. This model reliably disrupts all descending tracts and creates a uniform lesion cavity. Layered suturing was then performed to close the muscle and skin layers.

### 4.5. Transplantation Procedure

Immediately following SCI induction, rats were distributed across four treatment conditions. Animals exhibiting dural rupture/intraoperative death were immediately excluded. No other animals were excluded from the analysis. Animals were randomly assigned to the SCI, HGF, SVF, and SVF + HGF groups using a computer-generated randomization schedule. Animals were randomly allocated to the SCI, HGF, SVF, and SVF + HGF groups by simple randomization. The SCI group was only treated with a complete spinal cord crush without further treatment. To assess the individual contributions of the scaffold and the cells, one group (HGF) was administered a 10 μL injection of a 2% HGF hydrogel alone, while another group (SVF) received a 10 μL volume injection of an SVF cell suspension (2 × 10^4^ cells/μL) in HBSS. The experimental group (SVF + HGF) received the combinatorial therapy: initially, 5 μL of 2% HGF hydrogel was injected at the predetermined target site; subsequently, 5 μL of SVF cell suspension (2 × 10^4^ cells/μL) was delivered to the identical injection locus. Immediate post-injury transplantation was implemented using a Hamilton microsyringe (Cat. No. 1701; Hamilton, Bonaduz, Switzerland) with an attached glass micropipette. After careful puncture of the dura along the midline of the spinal cord, followed by insertion of the pipette to a 1.2 mm depth at the lesion epicenter. A total of 10 μL was injected slowly in two steps of 5 μL each, with a 5 min pause between injections to minimize backflow and ensure precise delivery. The wound was then covered with a piece of autologous abdominal fat to prevent leakage. Post-operatively, animals received subcutaneous gentamicin (2 mL, twice daily for 7 days). Manual urination was performed three times a day until bladder function recovered spontaneously after one week.

### 4.6. Behavioral Analysis

All outcome assessments—including behavioral tests (BBB scale and foot slip test), histological analyses, electrophysiological recordings, and proteomic data processing—were conducted by examiners fully blinded to the group allocation. The blinding was maintained until the completion of all data analyses.

BBB Locomotor Rating Scale: The BBB scale was used to assess hind limb motor function every day during the first week after SCI and transplantation, and weekly thereafter [56]. Two independent observers scored the left and right hind limbs, and mean scores served as analytical values.

Foot-slip Test: Rats were placed on an elevated grid walkway (Clever Systems Inc. Reston, VA, USA) [57]. The number of foot slips (partial or complete) during 50 consecutive steps was recorded. Sores were 1 (partial fault) if the hind limb slipped but did not touch the glass plate or 2 (full fault) if the hind limb slipped and touched the glass plate. The foot fault rate (FF%) was calculated as FF% = [(Number of Partial Footfault × 1) + (Number of Full Footfault × 2)]/50 × 100%.

Neuropathic Pain Assessment:

Mechanical Allodynia: The paw withdrawal threshold was determined using an ascending series of von Frey filaments (North Coast Medical Inc. Morgan Hill, CA, USA) to the plantar region of the hind paws [58]. After 30 min of adaptation in the test cage, hind paws were stimulated by von Frey filaments from 0.16 g to 26 g until withdrawal. Each stimulation lasted for 5 s, with stimulus intervals longer than 5 min. The threshold was defined as the intensity (filament weight) eliciting at least three withdrawal reactions in five consecutive stimulations.

Thermal Hyperalgesia: Paw withdrawal latency was measured using a radiant heat source (IITC Life Science, Woodland Hills, CA, USA) directed at the hind paw. Thermal hyperalgesia was measured by applying radiant heat stimulation between 30 °C and 50 °C to the sole of the hind foot. Each hindfoot was measured 5 times with an interval of 5 min, and the average duration of paw withdrawal was used as the paw withdrawal latency [59].

Electrophysiology: Fifty-six days after transplant treatment, rats were anesthetized by intraperitoneal injection of 1% sodium pentobarbital. Hair was removed from both eyes of rats up to the interoccipital and posterior thigh skin, and the surgical area was sterilized with iodophor. The rat head was fixed on a stereotaxic apparatus (Cat. No. 69101; RWD, Shenzhen, China). The anterior fontanel was used as the reference point, 2 mm down, and holes were drilled 1.5 mm on each side, with a skull opening size of 2 × 2 mm^2^. The stimulating electrode of the BL-420 biological function experimental system (Tme, Chengdu, China) was connected to the motor cortex of rats, and the receiving electrode was placed in the contralateral gastrocnemius muscle for single stimulation [5,60]. The stimulation voltage was 10 volts, the interval time was 0.1 s, and the detection time was 2 min. The latency and amplitude of each group were recorded.

### 4.7. Tissue Processing and Histology

At 14 and 56 days post-transplantation, rats received transcardial perfusion with 0.9% saline, followed by 4% paraformaldehyde (PFA). A 1.6 cm spinal cord segment that was centered at the lesion site was dissected and subsequently post-fixed in paraformaldehyde (PFA) for an overnight incubation and cryoprotected in a graded sucrose series (10%, 20%, 30%). Following embedding in OCT compound, the tissue was sectioned into 20 µm coronal and horizontal slices with a cryostat.

### 4.8. Immunofluorescence Staining and Quantification

Tissue sections were subjected to blocking treatment with 10% normal donkey serum, followed by an overnight incubation at 4 °C with the primary antibodies mouse anti-NeuN (1:1000, Cell Signaling Technology, Danvers, MA, USA), goat anti-ChAT (1:500, Millipore, Burlington, MA, USA), rabbit anti-NF200 (1:500, Sigma), mouse anti-Iba1 (1:1000, Wako, Osaka, Japan), and mouse anti-CD68 (1:500, Abcam, Cambridge, UK). Following washing, sections were incubated with corresponding Alexa Fluor-conjugated secondary antibodies (1:1000, Invitrogen, Grand Island, NY, USA; Abcam, Cambridge, UK) and counterstained with DAPI. Images were acquired using a fluorescence microscope (Carl Zeiss, Oberkochen, Germany). Double-blinded quantification of positive cells was implemented across specified regions of interest (ROIs), which were positioned at 1 mm intervals rostral and caudal to the lesion epicenter.

### 4.9. Data-Independent Acquisition (DIA) Proteomics

To further explore the potential molecular basis behind SVF + HGF transplantation benefits for SCI, the goal of this study is to identify and analyze the differentially expressed proteins among different groups and thus uncover the key molecular events through which this combined therapeutic strategy exerts its therapeutic efficacy. Spinal cord tissues (1 cm centered region at the lesion site) were harvested from the SVF + HGF, SCI, and normal groups (*n* = 3 per group) 14 days after SCI. Proteins were extracted, digested with trypsin, and analyzed by liquid chromatography-tandem mass spectrometry (LC-MS/MS) using a Q-Exactive HF mass spectrometer (Thermo Fisher Scientific, Waltham, MA, USA) in DIA mode. The raw data were processed and analyzed with Spectronaut 18.0 software. Differential expression analysis was carried out, and proteins with a fold change > 1.5 and *p*-value < 0.05 were considered significant. To further clarify the molecular regulatory mechanisms, we examined the enrichment of Gene Ontology (GO) terms and Kyoto Encyclopedia of Genes and Genomes (KEGG) signaling pathways. Quality control metrics included mass accuracy < 10 ppm, average identified peptides per run = 84,372, and average identified protein groups = 6809. FDR thresholds were set at <1% for both peptide and protein levels. A summary of peptide and protein identification counts across groups is provided in Supplementary Figure S2. Principal component analysis (PCA) was performed on the quantified protein expression matrix to visualize global protein expression differences among the groups. Data were mean-centered and scaled to unit variance prior to PCA, and the PCA score plot was generated based on the first two principal components (Supplementary Figure S3). The dataset identifier is PXD080155.

### 4.10. Statistical Analysis

Data are expressed as mean ± SD. Statistical significance was calculated using GraphPad Prism 8. An unpaired two-tailed Student’s *t*-test was used to compare two experimental groups. We first tested homogeneity of variances using a *t*-test and, if variances were significantly different, used a *t*-test with Welch’s correction. For comparison of more than two groups, one-way analysis of variance (ANOVA), followed by the Tukey post hoc test, was employed. The level of significance was set at 0.05.

## Data Availability

The datasets generated during the current study are available from the corresponding author on reasonable request.

## Supplementary Materials

The following supporting information can be downloaded at https://www.mdpi.com/article/10.3390/gels12070638/s1; Figure S1: CONSORT flow diagram illustrating the experimental allocation and grouping of female Sprague-Dawley (SD) rats subjected to T9 spinal cord crush injury; Figure S2: Overview of peptide and protein identification in Control, SCI and SVF + HGF groups, including peptide counts, protein counts per replicate, and the distribution of quantified proteins by sample detection coverage; Figure S3: Proteomic PCA analysis reveals obvious intra-group clustering and significant inter-group discrimination among the three sample groups; Table S1: Summary Table of Physicochemical and Functional Performance Comparison of HGF-RADA16-IKVAV Self-Assembling Peptide Hydrogel Materials; Table S2: Statistics of Animal Grouping and Sample Size for Each Experimental Item in Spinal Cord Contusion Rat Experiment.
