# Supplementary material for: SVF Combined with HGF-Functionalized Self-Assembling Peptide Hydrogel Promotes Spinal Cord Injury Repair in Rats
Source: Gels. 2026 Jul 16;12(7):638. doi: 10.3390/gels12070638 (PMC13409384; doi:10.3390/gels12070638)
Supplement: Supplementary file 1 [file gels-12-00638-s001.zip › gels-4372269-supplementary.pdf]

Supplementary Table S1. Summary Table of Physicochemical and Functional Performance Comparison of HGF-RADA16-IKVAV Self-Assembling Peptide Hydrogel Materials.

Performance Classification	Parameter Indicators	HGF-Functionalized Self-Assembling Peptide Hydrogel (Chen X et al., Gels, 2026)	HGF/pCNT Electroactive Nanohybrid Hydrogel (He L et al., ACS Appl Mater Interfaces, 2020)
Basic Formulation	Matrix Composition and Working Concentration	The matrix is HGF-modified RADA16 self-assembling peptide (sequence: RADA16-IKVAV-GG-HGF) with a working concentration of 2% (w/v).	The matrix is HGF-functionalized self-assembling peptide incorporated with polydopamine-modified carbon nanotubes (pCNT); the working concentration of the peptide is 1% (w/v), and the maximum loading capacity of pCNT reaches 13.7 wt%.
Storage Modulus (Rheological Mechanics)	Modulus Values and Gel Characteristics	The storage modulus of the composite hydrogel at a concentration of 1% is approximately 1.6 kPa; the mechanical strength is significantly improved after the introduction of pCNT; G' is consistently greater than G" within the linear viscoelastic region, stably maintaining an elastic gel state	
Nanostructured Fiber	Morphology and Dimensional Parameters	Three-dimensional porous nanofiber networks are formed via self-assembly; quantitative parameters such as fiber dimensions are cited from previous studies and not independently characterized in this work.	Uniform nanofibers are fabricated through self-assembly with a diameter of 25.3±2.3 nm, a length of 224.1±86.1 nm and a single-fiber thickness of 1.6±0.2 nm, which are rich in β-sheet secondary structures. pCNTs are homogeneously dispersed to construct interpenetrating hybrid networks.
Self-assembly/Gelation Kinetics	Gelation Conditions and Time	After adjusting the pH of the solution to 7.4 at room temperature, self-assembly completes within 30 minutes to form a stable hydrogel.	Exposure to physiological buffer (PBS) can rapidly trigger self-assembly; in-situ gelation occurs immediately after injection, forming an intact columnar structure.
Wet tissue adhesion performance	Tissue adhesion and fitting capacity	The material is designed as an intracranially injectable scaffold for cell delivery and repair, suitable for filling brain tissue cavities.	It features favorable injectability and filling performance, and is primarily applied to construct conductive microenvironments to regulate nerve regeneration.

Supplementary Table S2. Statistics of Animal Grouping and Sample Size for Each Experimental Item in Spinal Cord Contusion Rat Experiment

Experimental Project	SCI group	HGF group	SVF group	SVF+HGF group	Total
Behavioral	6	6	6	6	24
Histology（day 14）	3	3	3	3	12
Histology（day 56）	3	3	3	3	12
Electrophysiology	5	5	5	5	20
Proteomics	3	3	3	3	12
Total of single-group experimental animals	20	20	20	20	80

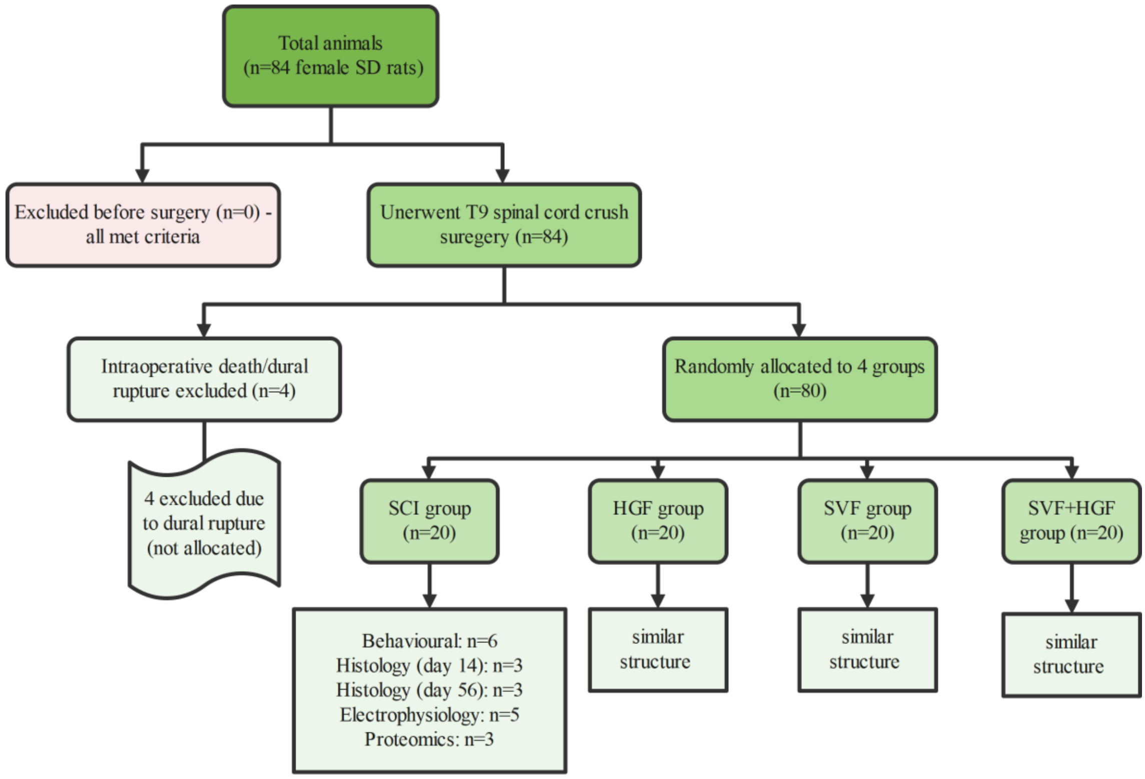

Supplementary Figure S1. CONSORT flow diagram illustrating the experimental allocation and grouping of female Sprague-Dawley (SD) rats subjected to T9 spinal cord crush injury. Of the initial 84 rats, 4 were eliminated intraoperatively due to death or dural tear, and the remaining 80 rats were randomly divided into four groups (n=20 each): SCI, HGF, SVF, and SVF+HGF groups. The detailed sample distribution for behavioural, histological, electrophysiological and proteomic assays in the SCI group is shown, with consistent sampling strategies adopted for all treatment groups.

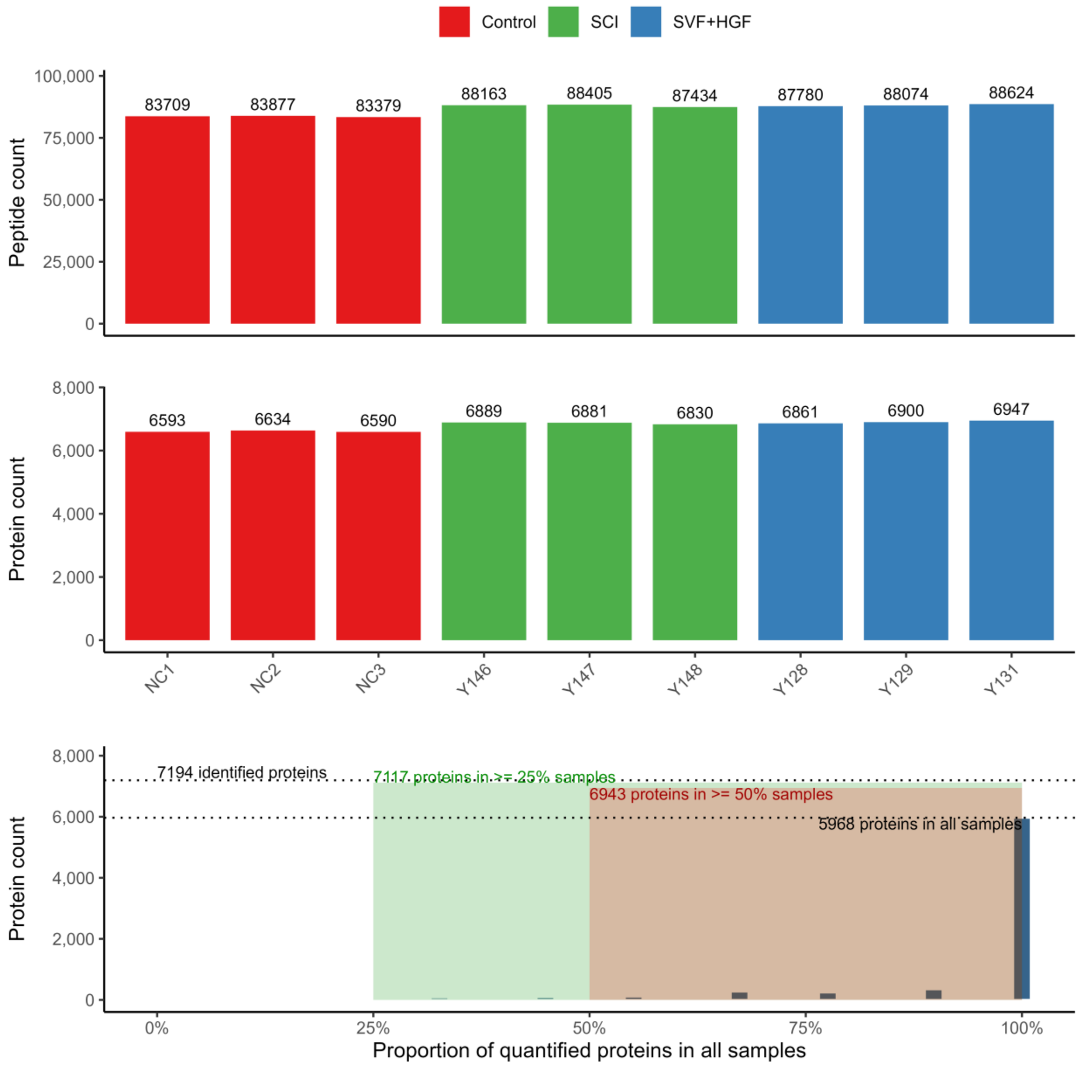

Supplementary Figure S2. Overview of peptide and protein identification in Control, SCI and SVF+HGF groups, including peptide counts, protein counts per replicate, and the distribution of quantified proteins by sample detection coverage.Data were deposited at ProteomeXchange (PXD080155).

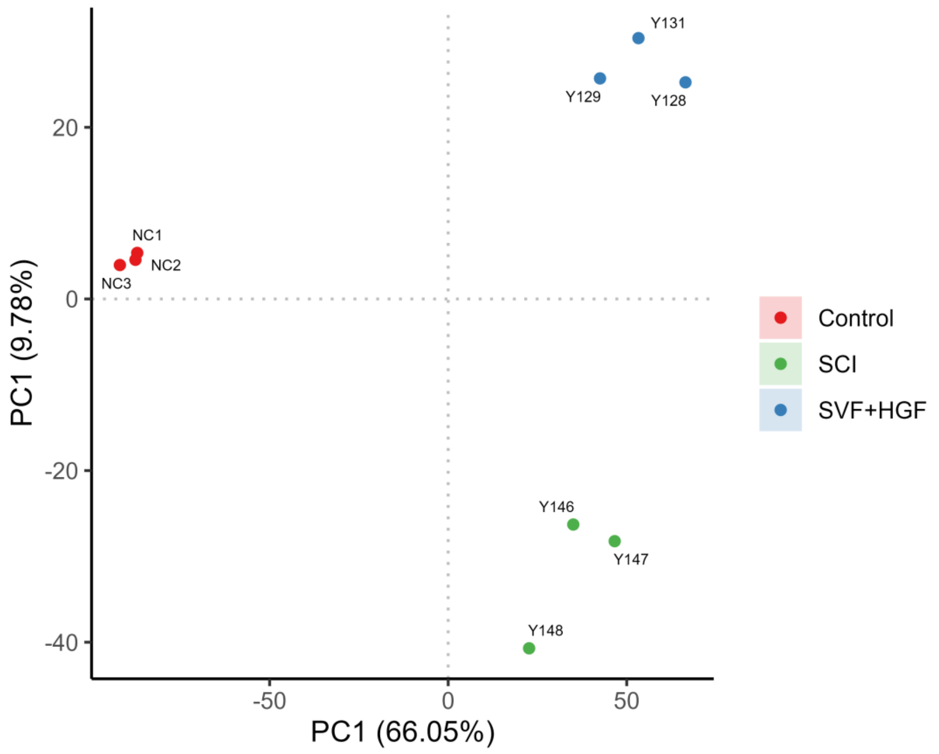

Supplementary Figure S3. Proteomic PCA analysis reveals obvious intra-group clustering and significant inter-group discrimination among the three sample groups. Data were deposited at ProteomeXchange (PXD080155).
